# A Note on the Depth-from-Defocus Mechanism of Jumping Spiders

**DOI:** 10.3390/biomimetics2010003

**Published:** 2017-03-14

**Authors:** Aleke Nolte, Daniel Hennes, Dario Izzo, Christian Blum, Verena V. Hafner, Tom Gheysens

**Affiliations:** 1 Bernstein Center for Computational Neuroscience Berlin, Unter den Linden 6, 10099 Berlin, Germany; 2 Robotics Innovation Center, German Research Center for Artificial Intelligence, 28359 Bremen, Germany; daniel.hennes@dfki.de; 3 Advanced Concepts Team, European Space Agency, 2200AG Noordwijk, The Netherlands; dario.izzo@esa.int; 4 Adaptive Systems Group, Department of Computer Science, Humboldt-Universität zu Berlin, Unter den Linden 6, 10099 Berlin, Germany; blum@informatik.hu-berlin.de (C.B.); hafner@informatik.hu-berlin.de (V.V.H.); 5 Polymer Chemistry & Biomaterials Research Group, Department of Organic and Macromolecular Chemistry, Ghent University, Krijgslaan 281 S4Bis, B-9000 Ghent, Belgium; tom.gheysens@ugent.be

**Keywords:** depth estimation, depth-from-defocus, computer vision, spider vision, spider eye

## Abstract

Jumping spiders are capable of estimating the distance to their prey relying only on the information from one of their main eyes. Recently, it has been shown that jumping spiders perform this estimation based on image defocus cues. In order to gain insight into the mechanisms involved in this blur-to-distance mapping as performed by the spider and to judge whether inspirations can be drawn from spider vision for depth-from-defocus computer vision algorithms, we constructed a three-dimensional (3D) model of the anterior median eye of the *Metaphidippus aeneolus*, a well studied species of jumping spider. We were able to study images of the environment as the spider would see them and to measure the performances of a well known depth-from-defocus algorithm on this dataset. We found that the algorithm performs best when using images that are averaged over the considerable thickness of the spider’s receptor layers, thus pointing towards a possible functional role of the receptor thickness for the spider’s depth estimation capabilities.

## 1. Introduction

**Figure 1:**
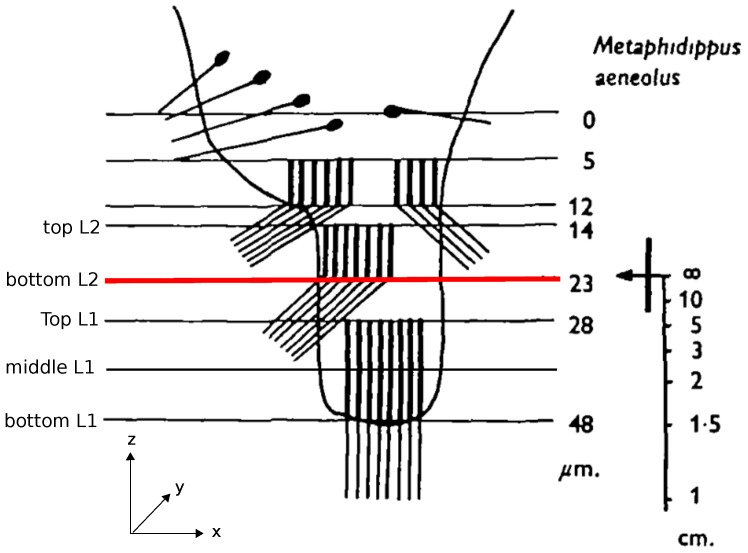
Arrangement of photoreceptor layers of *Metaphidippus aeneolus* anterior median eye’s retina. Light from the lens enters the figure from the top. The bar on the right indicates the distances (on the optical axis) at which objects would appear in focus on the corresponding horizontal planes on the retina. The red line highlights the location of the focal plane. The legend on the left indicates the location of the image planes used in our dataset. Adapted with permission from *The Journal of Experimental Biology* [2].

Jumping spiders are known for their impressive hunting behavior, in which they jump accurately to catch their prey. Recently, it has been shown that jumping spiders, more precisely *Hasarius adansoni*, estimate the distances to their prey based on monocular defocus cues in their anterior median eyes [1]. The retinae of these eyes have a peculiar structure consisting of photoreceptors distributed over four vertically stacked layers (Figure 1). Analyzing the optics of the eye and the spectral sensitivities of the photoreceptors, Nagata et al. [1] and Land [2] showed that only the bottom two layers, L1 and L2, are relevant for depth estimation (Figure 1). When an object is projected onto these two layers of the retina, the projected images differ in the amount to which they are in or out of focus due to the spatial separation of the layers. For example, if the image projected on the deepest layer (L1) is in focus, the projection on the adjacent layer (L2) must be blurry. Because of the achromatic aberrations of the spider’s lens, the light of different wavelengths has different focal lengths so that the same amount of blur on e.g., L2, could be caused by an object at distance *d* viewed in red light or the same object at a closer distance $d^{\prime }$ viewed in green light (cf. Figure 3 in [1]). In the spider’s natural environment, green light is more prevalent than red light and also the receptors in L1 and L2 are most sensitive to green light, therefore considerably less responsive to red light. In an experiment where all but one of the anterior median eyes were occluded, Nagata et al. could show that the spiders significantly underestimated the distance to their prey in red light, consistently jumping too short a distance, while performing the task with high accuracy under green light [1]. This means, that, in the red light condition, the spider must have assumed the prey to be located at the closer distance $d^{\prime }$, which would have been the correct estimation based on the blur in the more natural green light scenario. Accordingly, this also means that the amount of defocus in the projections onto L1 and L2 must be the relevant cue from which jumping spiders deduce distances. However, the underlying mechanism and neuronal realizations of how these defocus cues are transferred to depth estimates remain unknown. In computer vision (CV), however, this method of estimating distances from defocused images of a scene is well known under the term depth-from-defocus (DFD). The main idea of computer vision depth-from-defocus (CV-DFD) algorithms is to estimate the blur levels in a scene and then to use known camera parameters or properties of the scene to recover depth information. The first basic algorithms were proposed in the late 1980s and have been subsequently expanded upon in various ways, mainly for creating accurate depth maps of whole scenes [3,4,5].

In contrast to the spider eye which has a thick lens, CV-DFD is concerned with thin lenses or corrected lens systems. Projections through thick lenses show varying amounts of blur due to the lens’ spherical and achromatic aberration, while theoretical thin lenses or corrected lens systems result in less complex blur profiles. Therefore, some interesting questions arise: either the spider’s depth estimation mechanism is functionally different to the mechanisms underlying CV-DFD algorithms, or the CV-DFD algorithms are also applicable for the spider’s thick lens setup. In the former case, investigating the spider eye might result in findings which might inspire new algorithms which are also applicable for thick lenses.

For distance estimation in practice, a thick lens DFD is a promising technique. Particularly from a hardware perspective, a DFD sensor mimicking the spider eye setup by using only a single uncorrected lens and two photo sensors would be cheaper and more robust than the expensive and fragile corrected lens systems commonly used for DFD. In addition, when considering other widely used distance sensing equipment like laser detection and ranging systems (LADAR), sound navigation and ranging systems (SONAR) and stereo vision systems, (spider-inspired) DFD sensors seem worthy of advancing. Not requiring any mechanical or moving parts prone to malfunction, they would be much more robust and lower in maintenance. Due to the absence of moving parts and thus power-consuming actuators, energy would only be needed for the computational hardware resulting in a sensor that would be low in energy consumption. All of these properties would result in a sensor that would be cheap and, in turn, would allow for redundant employment or utilization as an assisting or fall-back option for other methods, making the sensor highly usable for unmanned aerial vehicles (UAVs), micro air vehicles (MAVs), and U-class spacecraft (CubeSats).

To investigate if we can learn from the spider for the creation of such a basic DFD sensor and to answer the question of whether CV-DFD algorithms are suitable for the spider’s thick lens setup, we created a computer model of the anterior median eye of the spider *Metaphidippus aeneolus*, a species for which the relevant parameters of the anterior median eye are well described in the literature [2]. For a structure as small and fragile as a spider eye, a computer model was preferred over an experimental approach, as experimental approaches have many more physical and experimental limitations rendering measurement of what the spider sees difficult, time-consuming and potentially inaccurate. In contrast, light tracing in optical systems is fully understood, meaning that results obtained from simulations are expected to be more accurate than measurements in an experimental setup. Using a generated computer model, we created a data set of images representing projections through the spider eye onto different locations on the retina and tested if a basic and well known CV-DFD algorithm [3], Subbarao’s algorithm, can estimate distances correctly based on these images.

## 2. Methods

### 2.1. Modeling the Spider Eye

The anterior median eye of *M. aeneolus* was modeled with the help of the three-dimensional (3D) graphics software Blender (version 2.76) [6]. To achieve physically accurate results of how light is refracted through a lens and posterior chamber, we chose LuxRender (version 1.5) [7], a sophisticated ray tracing algorithm instead of Blender’s built-in *Cycles* engine. LuxRender traces light according to mathematical models based on physical phenomena and is more suitable for geometrical optics simulations as those required in our study (for a brief discussion on render quality see Appendix A).

The anterior median eye of the spider *M. aeneolus* resembles a long tube with a very curved lens at one end and a boomerang-shaped four layer retina at the opposite end. The four retina layers extend over a range of 48 μm with distances between layers and layer thicknesses indicated in Figure 1. In the retina, the receptors are arranged in a hexagonal lattice, with a denser spacing closer to the optical axis and a coarser spacing towards the periphery. The spacing in layer L2 is overall coarser than in L1, and the minimum receptor spacing found in L1 was 1.7 μm.

#### 2.1.1. Modeling Lens and Posterior Chamber

**Figure 2:**
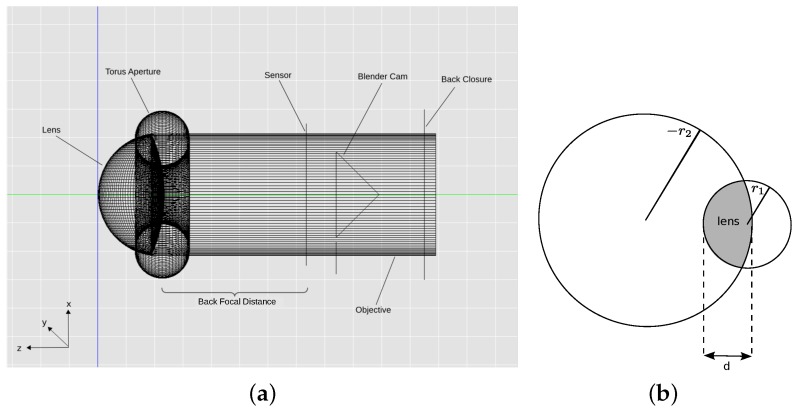
Eye model and lens parameters. (**a**) Blender model of the anterior median eye of *M. aeneolus*. The lens is a volume with refractive index n=1.41, the posterior chamber is a volume with refractive index n=1.335; (**b**) Illustration of lens parameters. $r_{1}$ and $-r_{2}$ are the radii of the front and back surfaces of the lens, respectively, *d* is the thickness of the lens.

In our model, the eye is represented by a thick lens enclosed by a black tube (Figure 2a). The thick lens has a back radius of $r_{1}$ = 217 μm, front radius of $r_{2}=-525\;\mu m$ and a thickness of d=236μm (illustrated in Figure 2b), and a refractive index of n=1.41.

The posterior chamber of the eye was modeled by setting the refractive index of the back of the lens to that of spider ringer, i.e., n=1.335. Aperture and specific shape of the lens (compare Land [2]) are achieved by creating a black torus with diameter $d_{torus}=200\;\mu m$ at the narrowest point. The resulting model is shown in Figure 2a. All the measurement values mentioned above are provided by Land [2] and summarized in Table 1.

#### 2.1.2. Modeling Receptor Layers/Sensor Spacing

For simplicity, we did not model the receptor layers as volumes (Figure 1), but simply as two-dimensional (2D) sensor planes. A sensor plane is realized in Blender by creating a plane of *translucent* material (to act as a “film”) and placing an orthogonal scene camera behind it to record the image on the film. To still capture the volume property in this simplified setup, we rendered several 2D sensor planes for each of the receptor layers. Nagata et al. [1] reported that the spider’s receptors are most sensitive to green light. In order to exclude confounds that would result from the lens’ chromatic aberrations, we illuminated the scene with a green light source and only used the image’s green channel.

We recorded images at locations of the sensor plane in the *z*-axis corresponding to the top of L2, the focal plane (which coincides with the bottom of L2), the top of L1, the middle of L1 and the bottom of L1. The corresponding back focal distances (BFD) are 450μm, 459μm, 464μm, 474 μm and 485 μm, respectively. In our setup, we chose a quadratic film size and based the number of receptors (=pixels, px) on the closest spacing found in L1 and L2, resulting in a 117 px × 117 px film of size 200 μm × 200 μm to approximate the receptors of a layer. We used this film to record images for L1 as well as for L2. While the receptors in the spider eye are distributed over a boomerang-shaped region (see Figure 5 in [2]), which extends over approximately 200 μm in the *x*-direction but only measures a few micrometers at its most narrow point in the *y*-direction, we assume that the spider can emulate a larger retina by moving the retina in the *x*–*y* direction, probably “stitching” the partial images together to form a larger image. This scanning behavior has been seen before [2] and might also result in higher visual accuracy, emulating a more dense receptor spacing. We thus modeled the retina as a square.

### 2.2. Data Set

We generated the data set by rendering 28 different images (“objects”) as projected onto the retina layers. We rendered projected images for each object placed at distances (D) from 0.9 cm to 3.1 cm, which is the same distance range that is used for the original experiments [1]. The distances 2.5 cm and 1.5 cm approximately correspond to the conjugate planes (in object space) to the middle of L1 and the top of L1 (see Figure 1), and thus “good” images, i.e., images that theoretically have low amounts of blur, are part of the data set.

**Figure 3:**
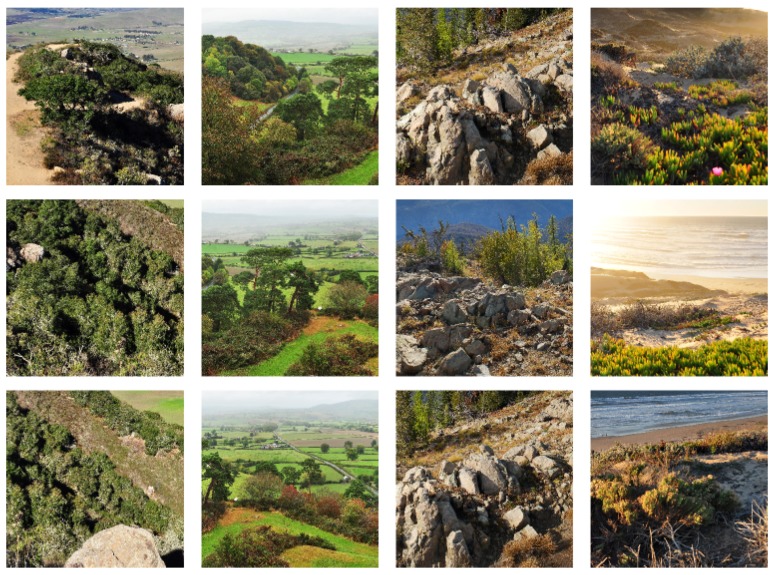
Examples of the images used to generate the dataset. These are the “objects” that are placed in front of the lens to be viewed through the lens. Images are 2000 px × 2000 px sub-images from landscape images by Gregg M. Erickson [8], available under the Creative Commons Attribution 3.0 License [9]. The complete data set of all 28 input images can be found in Figure A2.

**Figure 4:**
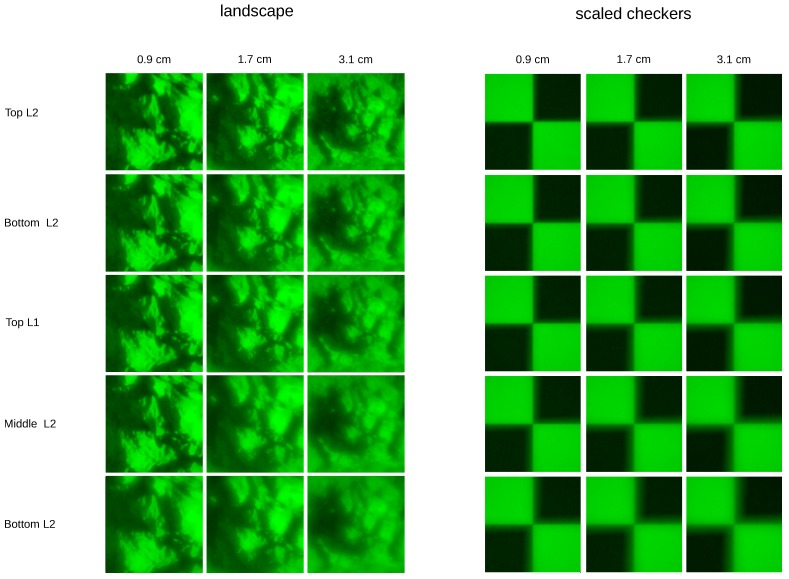
Examples of images as projected through the spider’s lens onto different layers of the retina (rows) and for different object distances (columns). Examples are shown for one of the landscape scenes and a checkerboard texture which is scaled with the object distance, in order to allow better visual inspection of blur. Resolution of the projections is 117 px × 117 px on a retina size of 200 μm × 200 μm.

The projected objects are a lens magnification-adjusted checkerboard texture (for easier visual inspection, not used in the experiments) and sub-images from large landscape images (Figure 3) with textures at different scales, for example trees, rocks and smaller plants. Examples for the projected landscape as well as the checkerboard textures are shown in Figure 4. Even though in the original scene the trees, rocks and plants might have been located at different distances, it is not these distances that the algorithm is supposed to recover, but the distance at which the image as a whole is located from the lens. In a single photograph, the depth information of the individual components is not preserved and the landscape is merely selected for the various scale textures, which ensures that the sub-images contain enough frequency content, which is a requirement for the DFD algorithm [3]. To calculate depth maps of 3D scenes, the procedure would be to split the images resulting from the projections onto the sensor planes into patches [5]. Under the assumption that the parts of the 3D scene which correspond to each patch are of constant depth, distances are then estimated for each pair of patches separately. Good depth recovery for 2D scenes is thus a prerequisite for depth maps of 3D scenes. However, 3D scene depth recovery suffers from the *image overlap problem* (cf. Section 2.3) and requires employing additional heuristics to get consistent depth maps. As the aim of this work is to find out if depth can, in principle, be recovered from images as the spider sees them, we perform our experiments only on the above described 2D planes.

### 2.3. Depth-from-Defocus Algorithm

In the following, we present the reasoning underlying DFD algorithms and describe a widely used algorithm as proposed by Subbarao in 1988 [3]. Even though the algorithm has been improved upon in many ways since it has been proposed (e.g., [5]), most improvements address the *image overlap problem*, the problem that, when segmenting an image into patches to estimate the distance of each patch, each patch is influenced by objects in neighboring patches due to the spread of the defocus. In our setup, however, the objects we consider are planes perpendicular to the optical axis so that the distances are constant over the whole image. Accordingly, these improvements are not expected to increase the performance in our scenario, so that only the original algorithm is used.

#### 2.3.1. Subbarao’s Depth-from-Defocus Algorithm

If an object is not in focus, the amount of blur in the image can provide information about the distance of the object. Given that we know the camera’s parameters, namely the focal length, the aperture and the lens to sensor distance, we can calculate the distance by basic geometry and Gaussian lens formula. Gaussian lens formula assumes a thin lens and relates the object distance and the focal length of the lens to the distance of the image in focus:

(1) $$\frac{1}{f}=\frac{1}{D}+\frac{1}{v}\;,$$

where *f* is the focal length, *D* is the distance of the object to the lens and *v* is the distance of the sensor to the lens.

The diameter *b* of the blur circle is related to the other camera parameters

(2) $$b=A\;v\;(\frac{1}{f}-\frac{1}{D}-\frac{1}{v})\;,$$

where *A* is the aperture. The actual observed blur circle radius *s* in pixels on the sensor then depends on the camera constant *ρ* (which maps the size of the projection to pixels and depends in part on the pixel resolution and in part on other camera properties)

(3) $$s=\rho \;\frac{b}{2}\;.$$

If the diameter of the blur circle is known, Equation (2) can easily be solved for the object distance *D*. Accordingly, the basis of most DFD algorithms, including Subbarao’s algorithm, is the estimation of blur from two or more defocussed images. The basic premise of the algorithm is that an out of focus image can be created from a sharp image by convolving the sharp image with a point spread function (PSF) that corresponds to the blur. For simplicity, the PSF is often assumed to be a two dimensional Gaussian

(4) $$h\left(x,y\right)=\frac{1}{2\pi \sigma^{2}}e^{-\frac{x^{2}+y^{2}}{2\sigma^{2}}},$$

with spread parameter *σ*. A blurred image g(x,y) can thus be obtained from a sharp image f(x,y) by convolving the sharp image with the PSF

(5) g(x,y)=h(x,y)*f(x,y),

where * is the convolution operator. Convolution in the spatial domain corresponds to multiplication in the frequency domain. Thus, when considering at least two blurry images in the frequency domain, the need for a sharp image can be eliminated:

(6) $$\begin{array}{c} G_{k}\left(\omega ,\nu \right)=H_{k}\left(\omega ,\nu \right)F_{k}\left(\omega ,\nu \right) \end{array}$$

(7) $$\begin{array}{c} \frac{G_{1}\left(\omega ,\nu \right)}{G_{2}\left(\omega ,\nu \right)}=\frac{H_{1}\left(\omega ,\nu \right)}{H_{2}\left(\omega ,\nu \right)}\;. \end{array}$$

Using the frequency space representation of Equation (4)

(8) $$H\left(\omega ,\nu \right)=e^{-2\pi^{2}\left(\omega^{2}+\nu^{2}\right)\sigma^{2}}\;,$$

this leads to

(9) $$\frac{H_{1}\left(\omega ,\nu \right)}{H_{2}\left(\omega ,\nu \right)}=\exp\left(-2\pi^{2}\left(\omega^{2}+\nu^{2}\right)\left(\sigma_{1}^{2}-\sigma_{2}^{2}\right)\right)\;.$$

As $\frac{G_{1}\left(\omega ,\nu \right)}{G_{2}\left(\omega ,\nu \right)}=\frac{H_{1}\left(\omega ,\nu \right)}{H_{2}\left(\omega ,\nu \right)}$, rearranging then allows for extracting the relative defocus

(10) $$\sigma_{1}^{2}-\sigma_{2}^{2}=-\frac{1}{2\pi^{2}}\frac{1}{\omega^{2}+\nu^{2}}log\frac{G_{1}\left(\omega ,\nu \right)}{G_{2}\left(\omega ,\nu \right)}\;.$$

In order to obtain a more robust estimation, the relative defocus is averaged over a region in frequency space

(11) $$C=\frac{1}{2\pi^{2}N}\sum_{\omega }\sum_{\nu }\frac{-1}{\omega^{2}+\nu^{2}}log\frac{G_{1}\left(\omega ,\nu \right)}{G_{2}\left(\omega ,\nu \right)}\;,$$

where $G_{1}\left(\omega ,\nu \right)\neq G_{2}\left(\omega ,\nu \right)$ and *N* is the number of frequency samples. (Note that in the above equations we matched the notation for the Fourier transform to the one used in Python’s software packages [10], and, thus, formulas differ from the original paper.)

It is then possible to solve for the blur of one of the images, e.g., $\sigma_{2}$, by solving the following quadratic equation:

(12) $$\left(\alpha^{2}-1\right)\sigma_{2}^{2}+2\alpha \beta \sigma_{2}+\beta^{2}=C$$

with

(13) $$\alpha =\frac{v_{1}}{v_{2}}$$

and

(14) $$\beta =\rho v_{1}\frac{A}{2}\left(\frac{1}{v_{2}}-\frac{1}{v_{1}}\right)\;.$$

Using the obtained $\sigma_{2}$ in Equations (3) and (2) allows for solving for the distance *D*.

#### 2.3.2. Curve Fitting

Subbarao’s algorithm assumes ideal lenses and a noise-free imaging setup. In order to still be able to estimate distances for the case that the relative defocus estimates (*C*-values, Equation (11)) follow the desired trend but are subject to linear transformations, i.e., are a shifted or scaled version of the theoretical *C*-values, we perform least-squares curve fitting to find the potential scaling factors and shift values. To this end, we use all estimated *C*-values for a particular combination of sensor planes and select scale parameter $\gamma_{0}$ and shift parameter $\gamma_{1}$ such that the sum of the squared distances between the theoretical *C*-values and the estimated *C*-values is minimized. The model *f* to be fitted to the data is thus

(15) $$f\left(C,\gamma \right)=\gamma_{0}\times C+\gamma_{1},$$

where *C* is the theoretical relative defocus as determined by camera parameters and object distance, with

(16) $$\begin{array}{c} C=\sigma_{1}^{2}-\sigma_{2}^{2}\; \end{array}$$

(17) $$\begin{array}{c} =s_{1}^{2}-s_{2}^{2}\; \end{array}$$

(18) $$\begin{array}{c} =\frac{rho^{2}}{4}A^{2}\left(\frac{1}{D^{2}}\left(v_{1}^{2}-v_{2}^{2}\right)+\frac{2}{D}\left(v_{2}^{2}w_{2}-v_{1}^{2}w_{1}\right)+v_{1}^{2}w_{1}^{2}-v_{2}^{2}w_{2}^{2}\right) \end{array}$$

and

(19) $$w_{1}=\frac{1}{f}-\frac{1}{v1}$$

and

(20) $$w_{2}=\frac{1}{f}-\frac{1}{v2}\;.$$

**Figure 5:**
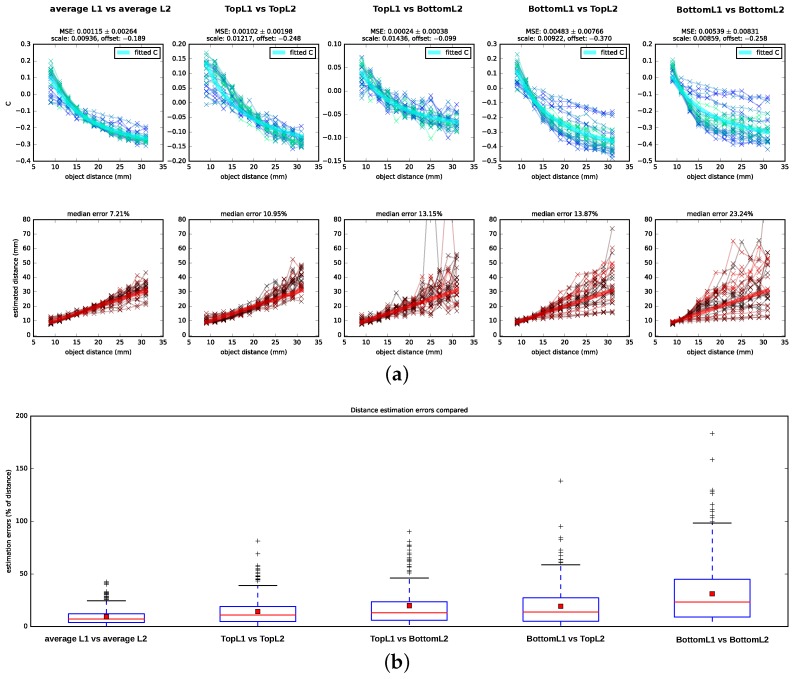
Performance of Subbarao’s algorithm on images generated with the spider eye model. (**a**) *C*-values and resulting depth estimates for Subbarao’s algorithm. Results are shown for images generated with the Blender spider eye model for all pairings of image planes, as well as a condition in which the planes available for each receptor layer are averaged, so that TopL2 and BottomL2 form an average representation for L2 and TopL1, MiddleL1 and BottomL1 form an average representation for L1. Connected data points of one color shade belong to projections of the same object (i.e., projections of images in Figure 3). For the *C*-values, the employed scale and offset parameters as well as the mean squared error (MSE) for the least-squares curve fitting procedure are reported on top of the subfigures. For the estimated distances, the corresponding ground truth distances are indicated by thick red lines. Plots are zoomed to the relevant sections and, therefore, some outliers are not shown; (**b**) Boxplot summarizing the distance estimation errors for the different conditions. Median errors are indicated by red horizontal lines, mean errors by red squares.

The *C*-value curves that result from this fit are depicted in Figure 5 as thick light blue lines.

## 3. Results

We tested Subbarao’s algorithm on the data set described in Section 2.2 by testing five different hypotheses of which retina planes might be used for the depth estimation: a combination of planes located at different depths of L1 and L2—namely, TopL1 vs. TopL2, BottomL1 vs. BottomL2, TopL2 vs. BottomL1, and BottomL1 vs. TopL2, (cf. Figure 1)—as well as an additional condition in which we averaged over all planes in a layer (TopL2 and BottomL2 vs. TopL1, MiddleL1 and BottomL1) and used the resulting average images as input for the algorithm (for details on image preprocessing refer to Appendix C). We showed the computed *C*-values (Equation (11)), including scaled and shifted ground truth *C*-values as determined by least square fitting (cf. Section 2.3.2), as well as the distances that are determined from the *C*-values and the camera parameters in Figure 5 alongside the corresponding ground truth values. Following the DFD literature, we reported the estimation errors for the distances in percentage of the ground truth distance. We found that the algorithm performs best on the averaged images with a median distance error of 7.21%. In addition, when using image pairs that are projected onto the surface planes, TopL2 and TopL1, the distance recovery is acceptable but considerably worse with a median squared error of 10.95%. For the other layer combinations, the distance estimation is less consistent: the number of outliers and hereby also the variances increase, and, particularly for distances further from the lens, the estimated *C*-values show a large amount of spread around the theoretical *C*-values.

## 4. Discussion

Subbarao’s algorithm yields good distance estimates (7.21% median error) on images that integrate the information available for each layer by averaging over the images formed on the top, (middle—only L1) and bottom receptor planes. For images formed on the surfaces of the receptor layers (TopL1 vs. TopL2), depths can still be estimated with a median error of 10.95%. For all other combinations of input layers, the *C*-values show a spread that is too large around the ground truth *C*-curve, which results in incoherent distance estimates.

The good performance in the two reported cases is surprising, as Subbarao’s algorithm assumes an ideal thin lens. A thick lens like in the spider eye might introduce strong artifacts due to spherical aberrations, like e.g., a variation of the blur strength dependent on the location relative to the optical axis, which could mislead the algorithm. It seems likely that the image planes for which the algorithm fails are located at distances relative to the lens where these aberrations have a more severe impact for most object distances and that the top surfaces of L2 and L1 are located relative to the lens and to each other such that the impact of the overall aberrations is either low or canceled out. If incoming photons are more likely to be absorbed towards the distal part of the receptors, it would make sense that the surfaces of the layers are located optimally instead of the middle or bottom of these layers.

However, the distance estimation on the averaged images is considerably more accurate than when using the surfaces’ layers. This may indicate that the considerable depth of the receptor layers (10 μm for L2 and 20 μm for L1) plays an additional role in depth estimation. Indeed, the receptor layer thickness might have a stabilizing effect, enhancing the depth estimation. When considering the performance in Figure 5 for TopL1 vs. TopL2 in more detail, the spread of the estimated *C*-values is higher for distances closer to the lens and varies least for object distances at about 2 cm. However, for the BottomL1 vs. the BottomL2 case, it is vice versa: the estimated *C*-values show a small spread for distances close to the lens and a very large spread for distances further away. Therefore, it may be possible that the thickness of the layers compensates in capturing the best suitable projection in both cases: towards the bottom of the layers for closer distances and towards the surfaces of the layers for intermediate and further distances.

Taken together, the spider’s depth estimation capabilities can be reproduced to a large extent by the mechanism underlying Subbarao’s algorithm. However, the estimated distances are still not perfectly accurate. This is likely due to noise in the algorithm, most likely in the calculation of the *C*-values, as the spectra are processed heuristically to discover the relevant values (shown in Appendix D) or due to noise in the rendering process. An indication that either algorithm or rendering are responsible was also shown in earlier experiments that we conducted with a thin lens model (see Appendix D), which resulted in a similar level of inaccuracy. Less likely, it may be that physiological parameters like lens shape and refractive indices may be used to construct the eye model are not completely accurate or that details in the physiology, like the exact spacing and layout of receptors, play additional functional roles. However, another possibility is that the spider’s performance could be even better explained by a more sophisticated DFD algorithm that is less sensitive to noise.

## 5. Conclusions

We presented images created by a model of the anterior median eye of the spider *M. aeneolus* and applied a well known DFD algorithm to these images. We found that the depth estimation capabilities of the jumping spider can be reproduced to a great extent by this algorithm, particularly when assuming that information is integrated over all of the receptor depths. We hypothesize that the latter might be an indicator that the receptor thickness plays a functional role in the depth estimation process.

## Figures and Tables

**Table 1:** Parameters of the anterior median eye of *M. aeneolus*.

Eye Parameters						
$r_{1}$	$-r_{2}$	**Aperture**	d	$n_{\mathrm{lens}}$	$n_{\mathrm{posterior}\;\mathrm{chamber}}$	f
217 μm	525 μm	200 μm	236 μm	1.41	1.355	504 μm

*d*: Lens thickness; *f* : Focal length; *n*: Refractive index; $r_{1}$: Radius of the front of the lens; $-r_{2}$: Radius of the back of the lens.

**Figure A1:**
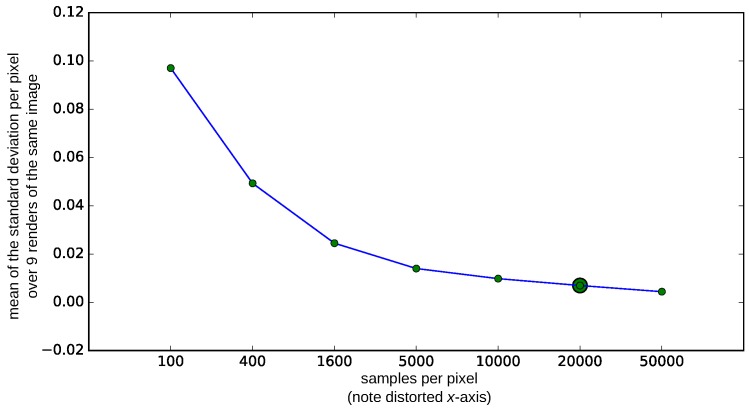
Render quality for different amounts of samples per pixel. The means of the standard deviations that are computed per pixel for nine different renders of the same image are shown. If the pixel values for the same pixel locations differ a lot, this indicates that the sampling is not sufficient and that the rendered image is still noisy. We rendered the images used in subsequent experiments with 20,000 samples per pixel (indicated by a larger marker), as for this value the rendered images are sufficiently similar and the rendering time is more feasible than for 50,000 samples per pixel.

**Figure A2:**
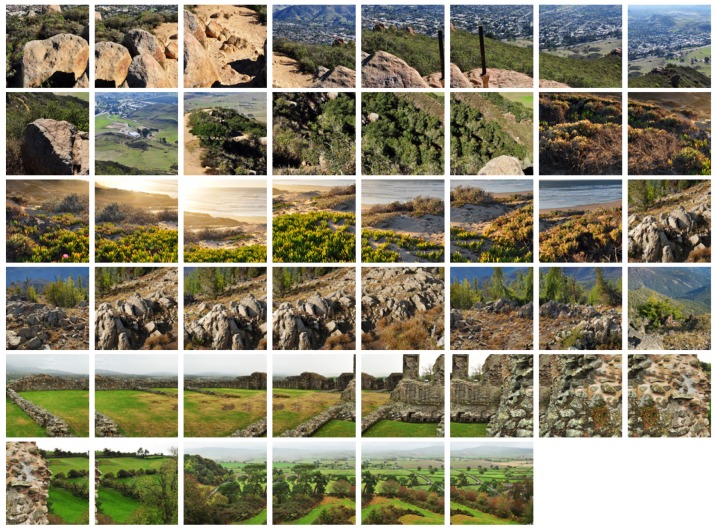
All input images used to generate the data set. Images are 2000 px × 2000 px sub-images from landscape images by Gregg M. Erickson [8], available under the Creative Commons Attribution 3.0 License [9].

**Figure A3:**
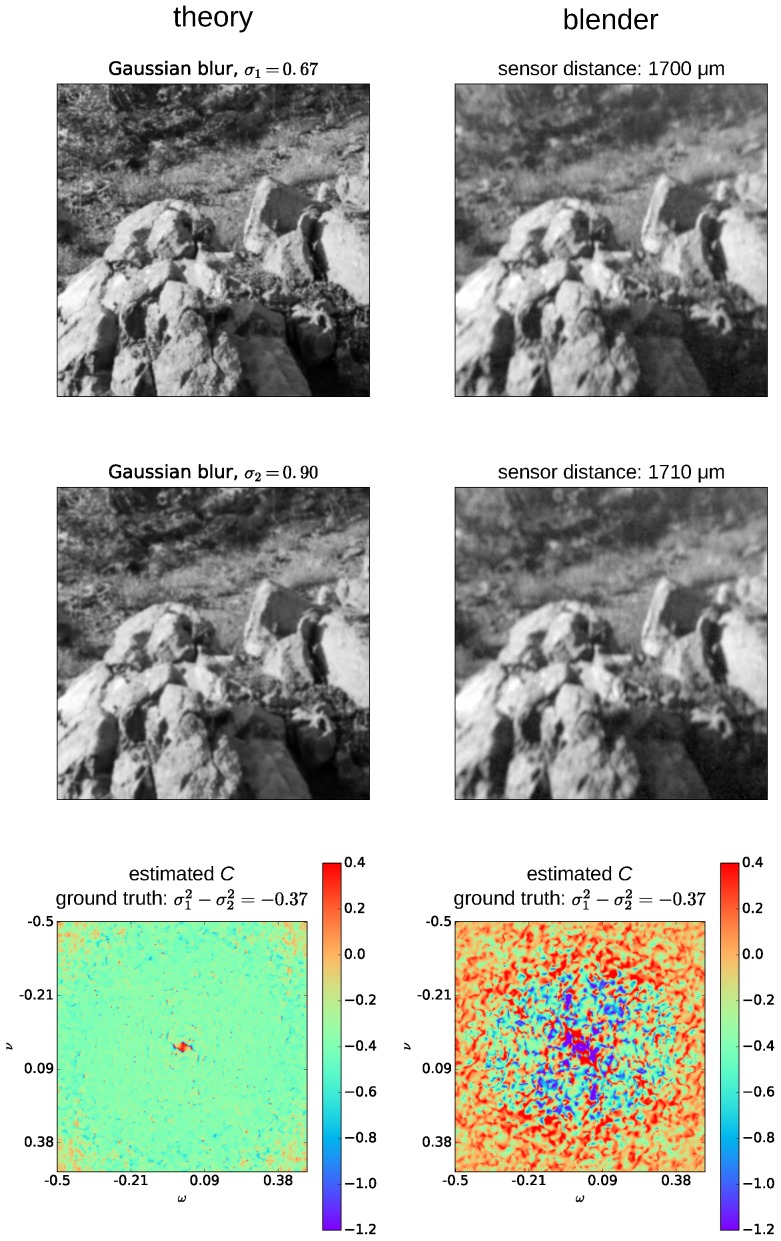
Inputs to Subbarao’s algorithm and the corresponding C-values for all frequency pairs (*ω* and *ν*) are shown. The left column of the figure shows how the inputs to Subbarao’s algorithm would look in the ideal case. The right column shows the images generated by the thin lens Blender model, and the object distance is 6 cm.

## Supplementary Materials

The Blender model, input images, resulting rendered images and analysis scripts are available on Zenodo: https://doi.org/10.5281/zenodo.377003.

## Abbreviations

The following abbreviations are used in this manuscript:

CV	Computer Vision
DFD	Depth-from-Defocus
CV-DFD	Computer Vision Depth-from-Defocus
L1	First Receptor Layer
L2	Second Receptor Layer
BFD	Back Focal Distance
PSF	Point Spread Function

## Appendix A. Render Quality

In preliminary experiments, we have found that the quality of the rendered image not only depends on the absolute number of light rays sampled per pixel, but also on the objects in the scene. As the spider eye model contains larger bodies of scattering and refracting materials like glass and water (lens and spider ringer), we ran an additional experiment to determine a suitable number of samples per pixel. Based on this experiment, we stop the rendering process when 20,000 samples per pixel are reached.

## Appendix C. Preprocessing

To dampen ringing artifacts that would be introduced by the borders of images, all projected images are windowed with a 2D Gaussian window with σ=0.1×image_width and subsequent zero-padding with a border of $\frac{image\_width}{2}px$ before applying the Fourier transform.

## Appendix D. C-Value Processing as Found by Thin Lens Experiments

As Subbarao’s algorithm failed in its unmodified setup on the spider images, we tested the algorithm first on a setup which is closer to the ideal thin lens that the algorithm assumes to test if additional processing was needed for the algorithm to work. To this end, we create another Blender model of a camera/an eye with a thin lens. The thin lens model is of the same configuration as the spider eye model as shown in Figure 2a, but with the dimensions of the components adjusted to the thin lens parameters (Table A1). To keep the thin lens model close to an ideal lens and to avoid dispersion effects, we set the material of the posterior chamber to air instead of water. Analog to the original data set, we create a new data set of the landscape images shown in Figure 3 as projected through this thin lens onto sensor planes at distances of 1700 μm and 1710 μm to the lens. Distances of the landscape images are from 2.5 cm to 7 cm from the lens in steps of 0.5 cm, and the sensor size and resolution corresponds with $\frac{117px\times 117px}{200\mu m\times 200\mu m}$ to the spider eye resolution.

**Table A1:** Parameters of thin lens.

Thin Lens Parameters						
$r_{1}$	$-r_{2}$	d	**Aperture**	$n_{\mathrm{lens}}$	$n_{\mathrm{posterior}\;\mathrm{chamber}}$	f
1000 μm	2000 μm	5 μm	156 μm	1.41	**Air**	1626 μm

*d*: Lens thickness; *f* : Focal length; *n*: Refractive index; $r_{1}$: Radius of the front of the lens; $-r_{2}$: Radius of the back of the lens.

Testing Subbarao’s algorithm on this data set reveals that distances can be estimated acceptably, but noise makes preprocessing of the estimated *C*-values necessary. This is illustrated in the bottom row of Figure A3, where the *C*-values for all frequency pairs (ω,ν) for ideal inputs, created by convolving a sharp image with the appropriate amount of Gaussian blur (left column), as well as for images generated by Blender (thin lens), are shown. It can be seen that, even for the ideal images, *C* is not as constant over frequencies as the formulation of Subbarao’s algorithm suggests. For example, the *C*-values in the leftmost plot show a disc-shaped region of values close to the desired *C*-value (bright green), while there is a lot of noise in the right plot. To make the estimate of *C* more robust against noise, we calculate the final *C*-value as the mean of the values between the 25 and 75 quantiles.
